# Phenotype–environment mismatch in metapopulations—Implications for the maintenance of maladaptation at the regional scale

**DOI:** 10.1111/eva.12833

**Published:** 2019-07-25

**Authors:** Jorge Octavio Negrín Dastis, Russell Milne, Frédéric Guichard, Alison Margaret Derry

**Affiliations:** ^1^ Départment des sciences biologiques Université du Québec à Montréal (UQAM) Montréal Québec Canada; ^2^ Groupe de recherche interuniversitaire en limnologie et en environnement aquatique (GRIL) Montréal Canada; ^3^ Department of Biology McGill University Montréal Québec Canada

**Keywords:** (a)symmetric selection, dispersal, heterogeneous landscapes, maladaptation, metapopulation, phenotype–environment mismatch

## Abstract

Maladaptation is widespread in natural populations. However, maladaptation has most often been associated with absolute population decline in local habitats rather than on a spectrum of relative fitness variation that can assist natural populations in their persistence at larger regional scales. We report results from a field experiment that tested for relative maladaptation between‐pond habitats with spatial heterogeneity and (a)symmetric selection in pH. In the experiment, we quantified relative maladaptation in a copepod metapopulation as a mismatch between the mean population phenotype and the optimal trait value that would maximize mean population fitness under either stable or fluctuating pH environmental conditions. To complement the field experiment, we constructed a metapopulation model that addressed both relative (distance from the optimum) and absolute (negative population growth) maladaptation, with the aim of forecasting maladaptation to pH at the regional scale in relation to spatial structure (environmental heterogeneity and connectivity) and temporal environmental fluctuations. The results from our experiment indicated that maladaptation to pH at the regional scale depended on the asymmetry of the fitness surface at the local level. The results from our metapopulation model revealed how dispersal and (a)symmetric selection can operate on the fitness surface to maintain maladaptive phenotype–environment mismatch at local and regional scales in a metapopulation. Environmental stochasticity resulted in the maintenance of maladaptation that was robust to dispersal, but also revealed an interaction between the asymmetry in selection and environmental correlation. Our findings emphasize the importance of maladaptation for planning conservation strategies that can support adaptive potential in fragmented and changing landscapes.

## 1 INTRODUCTION

Evolutionary principles are increasingly considered in the conservation of fragmented populations (Carroll et al., 2014; Hendry et al., 2011). Much of this focus has been on conditions that promote local adaptation as in for example stable or growing populations (e.g., Kawecki & Ebert, 2004; Kirkpatrick & Barton, 2006; Yeaman, 2015; Hoban et al., 2016). Less focus has been placed on maladaptation, in which population fitness is suboptimal and declining (Brady et al., 2019; Crespi, 2000; Hendry & Gonzalez, 2008). This is despite that maladaptation has the potential to influence metapopulation dynamics in fragmented habitats (Farkas, Mononen, Comeault, & Nosil, 2016; Marshall, Monro, Bode, Keough, & Swearer, 2010; Nicolaus & Edelaar, 2018). Maladaptation also appears to be promoted in human‐disturbed environments (road salt: Brady, 2013; oil spill: Rolshausen et al., 2015; climate change: Zimova, Mills, & Nowak, 2016; diversity of human contexts: Hendry, Gotanda, & Svensson, 2017). However, studies that have explicitly sought to establish expectations for the frequency and persistence of maladaptation in natural systems in relation to spatial structure and environmental fluctuations are rare (Laine, 2004; Lemoine, Doligez, & Richner, 2012; Tack, Horns, & Laine, 2014).

Maladaptation is widespread in natural populations (Brady et al., 2019) and evolutionary traps (Robertson & Chalfoun, 2016; Schlaepfer, Runge, & Sherman, 2002), inbreeding depression (Frankham, 2015), and phenotype–environment mismatch (Hendry et al., 2011; Zimova et al., 2016). Under phenotype–environment mismatch, relative maladaptation at the population level can be measured as a deviation of the mean trait value (and/or variance) and would only be present if fitness of the mean trait value is lower than the fitness achieved by the optimal trait value. Absolute maladaptation would only result if mean population fitness of the trait value declined below replacement. Maladaptation from phenotype–environment mismatch occurs when an organism that is adapted to one environment incurs a reduction in fitness in an alternative environment (DeWitt & Yoshimura, 1998), as a result of organism dispersal and/or environmental variation. The fitness surface (Schluter & Nychka, 1994) depends on the relationship between the fitness and the trait distribution, and both selection and the fitness function can be symmetric or asymmetric. The asymmetry of the fitness function is a direct consequence of the distribution of selection strength around the optimal trait and has usually been assumed to be symmetric under directional selection. A symmetric fitness surface means that fitness is a function of the trait deviation, independently from the direction of that deviation. In contrast, an asymmetric fitness surface depends on both the magnitude and direction of trait deviation from their fitness optimum. An asymmetric fitness adaptive trade‐off was previously documented in a natural system of copepods where acid‐adapted copepod individuals incur a lower cost in survival under circumneutral conditions than circumneutral‐adapted copepod individuals under a range of acidic to mildly acidic environmental conditions (Derry & Arnott, 2007; Negrín Dastis & Derry, 2016). Here, we use survival to low pH to quantify acid tolerance as a trait, and more specifically as an adaptive trait‐based response to (a)symmetric selection from low pH. Although fitness could not be directly assessed from our experiments, we provide an explicit function relating trait value (acid tolerance) to fitness in our model. We tested for evidence of an asymmetric fitness trade‐off in relation to pH in natural copepod populations and used our metapopulation model to study how this local fitness surface asymmetry interacts with regional symmetric dispersal and with overall selection strength to predict the magnitude and spatial distribution of maladaptation (*Z*) (see Methods section for a quantitative definition). Relative maladaptation is quantified on a trait space as any deviation in local fitness that is <1*SD* away from the global optimum. Absolute maladaptation is quantified when mean population fitness of the trait value declines below replacement. Asymmetric fitness surfaces (Figure S4) are common in various natural populations: habitat‐dependent asymmetric selection despite high levels of gene flow (Hoekstra, Drumm, & Nachman, 2004), adaptive reversals in acid tolerance at both local and regional spatial scales (Derry & Arnott, 2007), asymmetric selection and dispersal on the evolution of disease resistance (Munroe, Powell, Ford, Hofmann, & Klinck, 2015), and local divergence in copepod acid tolerance at the landscape level (Negrín Dastis & Derry, 2016). However, metapopulation models have mostly considered symmetric selection in addressing the persistence of maladaptation (Bolnick & Nosil, 2007; Hanski, Mononen, & Ovaskainen, 2010; Ronce & Kirkpatrick, 2001). To our knowledge, only a handful of studies have integrated asymmetric selection in metapopulation models (Munroe et al., 2015; Urban, Bürger, & Bolnick, 2013).

Maladaptation from phenotype–environment mismatch can occur under several different scenarios in natural populations. Phenotype–environment mismatch can occur in populations if the rate of changing environmental conditions exceeds the rate of phenotypic tracking (Pease, Lande, & Bull, 1989), such that the optimal population phenotype becomes a “shifting target” (Siepielski et al., 2009; Brady et al.,^2019^). However, gene flow, especially at low levels and operating in a local isolated patch before migration, can facilitate adaptive responses to selection by providing a source of adaptive variation and by reducing the arrival of maladapted alleles (Garant, Forde, & Hendry, 2007; Richardson, Brady, Wang, & Spear, 2016). On the other hand, high levels of migration may promote phenotype–environment mismatch in the recipient population if the migrants are from other locally adapted populations (Bolnick & Nosil, 2007; Farkas et al., 2016; Lenormand, 2002). Maladaptation can theoretically be maintained through a combination of selection for traits that are suboptimal in poor quality habitats (sinks), and migration between habitats with strong opposing selection (Farkas, Hendry, Nosil, & Beckerman, 2015; Urban & Skelly, 2006). More recent empirical studies (Brady, 2017; Cenzer, 2017; Jacob et al., 2017; Nosil et al., 2018) and models (Nicolaus & Edelaar, 2018) have highlighted the potential importance of directional rather than random dispersal in local adaptation and speciation. A better match between the individual phenotype and the environment, with natural selection theoretically driving evolution around a dynamic equilibrium, may vary depending on different mechanisms that enable organisms to cope with environmental heterogeneity. Symmetric versus asymmetric fitness surfaces to selection in phenotype–environment mismatch may provide a novel and understudied mechanism affecting local population growth. Over regional scales, dispersal among heterogeneous habitats could interact with asymmetric selection to affect the persistence of maladaptation in metapopulations.

Our study addresses knowledge gaps in expectations for the frequency and persistence of population maladaptation from phenotype–environment mismatch in relation to spatial structure and environmental fluctuations (Laine, 2004; Lemoine et al., 2012; Tack et al., 2014). A better understanding of the influence of these fluctuations on a character that changes in magnitude and direction through time seems central to appraisals of survival through rapid environmental change and to the concept of fitness itself (Simons 2009). We address how symmetric versus asymmetric fitness surfaces to selection in phenotype–environment mismatch influence the persistence of phenotypes found away from the optimal environmental value and negative population growth in populations inhabiting a landscape of fragmented habitats. For instance, asymmetric selection will result in different degrees of maladaptation depending on the direction of the trait mismatch. We report results from a common garden field experiment and from a metapopulation model that tested for the existence of an asymmetric fitness to low pH in copepod populations and predicted its importance for the maintenance of total regional maladaptation (expressed as the sum of local maladaptation values measured in each habitat) to pH in relation to spatial structure and environmental fluctuations. The field experiment tested the influence of interannual differences in regional selection from pH on phenotype–environment mismatch in a copepod metapopulation that occurs in a system of fragmented freshwater ponds that are subject to stable or fluctuating pH between years. The ponds are dominated by a single calanoid copepod species, *Leptodiaptomus minutus* Lilljeborg, which is known to locally adapt to lake or pond‐water pH over short spatial distances (Derry & Arnott, 2007; Negrín Dastis & Derry, 2016). In this particular copepod species, the fitness surface to selection can be asymmetric depending on water pH: Neutral pH‐adapted phenotypes are more strongly selected against in acidic water (3.6 ≤ pH ≥ 5.9; low survival of neutral pH‐adapted copepods to acidic water) than acidic pH‐adapted phenotypes in circumneutral water (pH ≥ 6.0; high survival of acid pH‐adapted copepods to circumneutral water) (Derry & Arnott, 2007; Negrín Dastis & Derry, 2016). Our hypothesis was that maladaptive acid tolerance in local copepod populations would be influenced by interannual differences in the pH conditions of the surrounding landscape (regional selection) through between‐pond migration and the asymmetric fitness of copepod population acid tolerance to pond pH. Phenotype–environment mismatch was anticipated to occur when the phenotypic composition of copepod populations in local ponds was more strongly determined by the phenotypic composition of migrants because of weak local selection (circumneutral pH), especially when regional selection was strong (i.e., acidic landscapes with strong selection for acid‐tolerant phenotypes). We predicted that this maladaptation would be absent in pond populations in which local selection against maladapted phenotypes was strong, because of low survival of neutral pH‐adapted phenotypes to acidic pH. However, the interpretation of such experiment currently lacks an integration of the range of dispersal over which we would expect maladaptation to be coupled at local and regional scales through evolutionary (maladaptation) and ecological (abundance distribution) variation at the landscape level. Understanding the role of dispersal is key to interpreting experimental results from a metapopulation perspective.

In the metapopulation model, our goal was to disentangle the interacting effects of between‐patch dispersal, (a)symmetric selection to low pH in the phenotype × selection (pH) interaction, and environmental fluctuations, on maladaptive phenotype–environment mismatch and population demography of the copepods. To do this, we extended a two‐patch metapopulation model (Ronce & Kirkpatrick, 2001) to formulate an (a)symmetric selection function that depends on both the magnitude and direction of population trait deviation from the optimum. We assumed that population fitness correlated with copepod acid tolerance, a trait that we selected for study because of local‐scale maladaptation and asymmetric fitness along pH gradients (Derry & Arnott, 2007; Negrín Dastis & Derry, 2016). We had three main objectives: (1) to examine the conditions under which local maladaptation was maintained in terms of interactions between selection strength, asymmetry of the fitness surface to low pH, and level of migration between populations; (2) to study the role of an evolutionary process (local pH selection) for the maintenance of regional maladaptation through its interaction with an ecological property (high regional growth and net connectivity of acid‐adapted individuals); (3) to examine the robustness of this eco–evo relationship (evolution of acid tolerance shaping population abundance), to spatially (un)correlated stochastic fluctuations in local pH.

**Figure 1:**
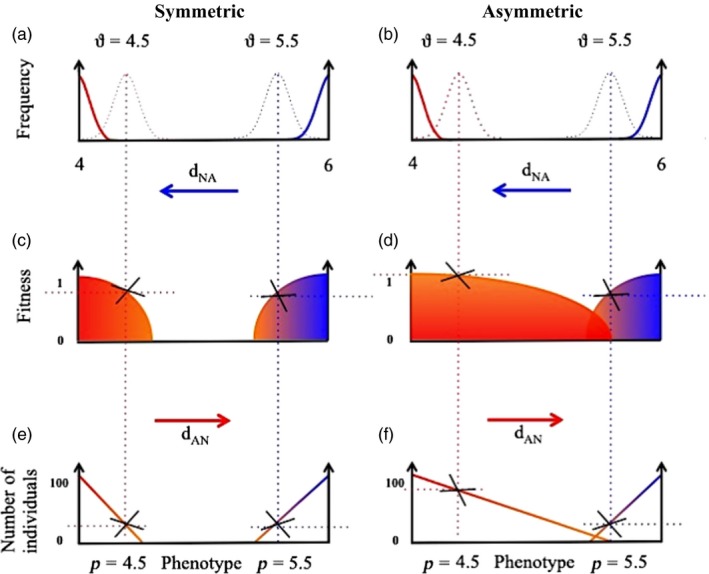
Conceptual figure introducing (a,b) the phenotypic frequency distribution of two spatially structured discrete populations exchanging individuals via bidirectional dispersal between patches: a circumneutral pH source copepod population (blue) and an acidic pH source copepod population (red). (c,d) symmetric versus asymmetric fitness surface to selection (evolutionary), (e,f) the influence of the fitness surface to selection on the number of individuals in the population (ecological). Crosses indicate hypothetical inflexion points as in (c) where fitness is suboptimal faraway from the optimum environmental threshold and (e) population sizes decline homogeneously across both patches. (d) A case of phenotype mismatch, in which population fitness and (f) population sizes decline heterogeneously across both patches. Please see Table 1 for a complete list of parameters

In our model, we anticipated that the symmetric fitness surface to low pH (Figure 1c) would bring population maladaptation and size to an equilibrium that was homogenous over the metapopulation (Ronce & Kirkpatrick, 2001). However, under a scenario of asymmetric fitness surface to low pH (Figure 1d), we predicted that weak selection would interact with limited dispersal and environmental fluctuations to increase both ecological (population growth) and evolutionary (maladaptation) effects. We refer here to an eco‐evolutionary process in the model because evolutionary change in a trait (acid tolerance) alters an ecological attribute (i.e., population abundance) through high regional growth and net connectivity of acid‐adapted individuals. Thus, pH acts as an agent of selection because it determines which individuals do or not tolerate directional selection from acidic pH, with per capita growth rates allowing for more abundant individuals depending on the source and destination of individuals across a heterogeneous two‐patch metapopulation. Our model shows how such eco‐evolutionary patterns can predict the dynamics of maladaptation that we detected in our field experiment. Our study highlights the importance of integrating a diversity of phenotypic responses of populations to spatial heterogeneity and environmental change for biodiversity conservation in fragmented landscapes. Our findings support conserving for functional traits and intraspecific trait diversity in metapopulations, rather than the present focus of conserving solely for species diversity.

## 2 METHODS

### 2.1 Empirical field experiment

The field transplant experiment was conducted in a “common garden” pond with copepods from isolated freshwater ponds subject to stable or fluctuating pH between years at Cape Race NL, Canada (46°38′33.35′′N, 53°12′02.27′′W). Although this landscape is predominately comprised of acidic (3.6 ≤ pH ≥ 5.9) ponds, an infrequent number of ponds have pH that remains ≥6.0 (circumneutral pH) between years and another infrequent category of ponds has pH that can fluctuate between acidic and circumneutral between years (Figure S1), depending on the level of springtime (April–May) precipitation (average precipitation hourly data 2013:16.25 ± 9.26 mm; average precipitation hourly data 2014:28.1 ± 5.8 mm, Environment and Climate Change Canada) that drains through the surrounding acidic peatlands into the ponds. The selection of ponds for our experiment was based on earlier samples taken which showed that *L. minutus* calanoid copepods where the most abundant prey species in this pond metapopulation system; therefore, interspecific competition for resources is unlikely. This pattern of spatial and temporal environmental heterogeneity between ponds, all located within 1 km and without surface water connections for the study ponds, enabled us to test differences in the influence of migrant copepod phenotypes on local copepod maladaptive acid tolerance, under similar dispersal, between years. The experiment was done in the summers of each of two years, 2013 and 2014, which had contrasting interannual average regional pond pH that could be used to reflect differences in the influence of the migration load of copepod phenotypes from the surrounding landscape of ponds on the average local copepod population phenotype.

The copepods were placed in translucent 20 L bottles and incubated under common garden environmental conditions (controlling for temperature, light, food quality, and presence of predators) for 7 days. We did exclude predators from the bottles by screening each sample through a 54 µm mesh right from the beginning of the experiment. The bottles were incubated in the same pond (pond below Q, with a surface area of 272 m^2^) and at the same depth (0.5 m) in each of two summers (June 2013 and June 2014). We used fixed levels for each factor as blocks with data from a factorial experiment. The factorial design for this experiment consisted of three fixed factors, 2 levels (Factor 1—Year; 2013, 2014) × 3 levels (Factor 2—Copepod population source; temporally stable circumneutral pH, temporally stable acidic pH and fluctuating pH) × 2 levels (Factor 3—pH treatment; pH 6.0, pH 3.6). We coded pond copepod source as a random variable in the model. We could quantify maladaptation in the experiment because the trait value and fitness are correlated, which let us use the trait as a proxy for fitness (the trait being defined as pH value leading to maximum survival). Since the landscape orientation and location of ponds, as well as distance between ponds, did not change between years and we do not have a measure of the genetic structure of the copepod metapopulation in this study system or empirical measures of rates of between‐pond dispersal on the landscape, we assumed that passive dispersal rates of copepods between ponds were similar between these years. Experimental design information and other methodological considerations can be found in the Appendix S1.

### 2.2 A metapopulation model with selection associated with strength, asymmetry in the fitness surface to low pH, and bidirectional dispersal

We used a 2‐patch metapopulation model based on Ronce and Kirkpatrick (2001) for the discrete‐time dynamics of local copepod populations within ponds connected by copepod dispersal and with between‐pond heterogeneity in pH.

#### 2.2.1 Local selection

We assumed pH is the only selection pressure operating on a single quantitative trait (pH tolerance), and we first consider the simplest case of a constant environment as defined by Lande and Shannon (1996), with stabilizing selection acting within generations, where the mean phenotype evolves to the optimum. As in other models (Falconer, Mackay, & Frankham, 1996; Lande, 1976), the rate of local evolution in the mean phenotype in response to selection is proportional to the product of the additive genetic variance and the intensity of directional selection. The local optimum pH in each local pond is expressed by the parameter *ϑ*, with population fitness reaching a maximum when the population average phenotype P is at the optimum (P = *ϑ*), which corresponds to local adaptation. We fixed *ϑ* = 4 and *ϑ* = 6 for our acidic and circumneutral pond, respectively. Selection strength Ɣ measures the intensity of stabilizing selection around this optimum. We implement our asymmetric function in the model as an additive cost contributing to total stabilizing selection, and controlled by the amount of symmetry α, with *α* = 0 and *α* = 0.5 corresponding to symmetric and asymmetric selection respectively (Figure 1). Further assuming homogeneous carrying capacity between ponds, the expected Malthusian fitness per generation of an individual with phenotype P at time *t* in a population of density *N* is (see Table 1 for a complete list of parameters):

(1) $$r=\text{ro}\left(1-\frac{N_{i}}{K}\right)-\gamma \left(\frac{{\left({\text{P}}_{i}-\vartheta_{i}\right)}^{2}}{2}\right)+\alpha \gamma \left(\frac{{\left({\text{P}}_{i}-\vartheta_{i}\right)}^{2}}{2}\right)\left({\text{P}}_{i}-\vartheta_{i}+\alpha \right)\left(\vartheta_{i}-\text{P}-\alpha \right)$$

**Table 1:** Two‐patch model parameters, brief description, and values used during simulations

Parameters	Description	Values
Local habitat dynamics:		
*ϑ*	Environmental optimum	(4,6)
P	Local phenotype	(4,6)
*N*	Initial number of individuals in the population	(100)
*K*	Carrying capacity	(1,000)
Spatial component:		
*d* a	Dispersal strength	(0, 0.1, 0.2, 0.3, 0.4, 0.5)
Local adaptation:		
Ɣ	Selection	(0.1, 0.2, 0.3, 0.4, 0.5)
*α*	Degree of asymmetry	(0, 0.5)
σp^2^	Phenotypic variance	(0.01)

a Acid disperses to neutral patch (*d*_AN_). Neutral disperses to acid patch (*d*_NA_).

Equation 1 refers to individual fitness of a specific phenotype P*_i_* in patch *i*. The first term in the right‐hand side of (Equation 1) describes per capita logistic growth, with ro the fitness at low density of an individual with the optimal phenotype, and *K* the maximal number of adapted individuals. We used the terminology and symbols used by Ronce and Kirkpatrick (2001) with the threshold fitness value for positive growth found at **r** = 0, because fitness is multiplied by density to calculate the change in population size at the next time step. Our population growth equation is of the form *Nt* + 1 = *Nt* + r *Nt*, with **r** > 0 leading to positive change in population size. The second term is zero here and only applies to (Equation 2a) representing population level growth and selection. The third term represents the *evolutionary load*, which is the difference between the optimum and the phenotype and describes mortality caused by stabilizing selection on the phenotype P (Lande & Shannon, 1996). The last term represents the asymmetric function and relates the trait value (acid tolerance) to fitness.

#### 2.2.2 Dispersal

Given the difficulty of gaining dispersal estimates for microscopic organisms such as zooplankton (Bilton, Freeland, & Okamura, 2001), the range of dispersal that was explored in our model, which was an interactive term with regional and local (a)symmetric selection, provides a picture of the parameter space over which we might expect to observe differences in maladaptation across a range of dispersal levels. This is especially important considering that even with similar rates of dispersal between local ponds in each given year, as we have assumed, the overall regional migrant load of maladapted phenotypes into local habitats could differ depending on the regional landscape context and frequency of acidic habitats across the landscape in a given year. Dispersal can introduce individuals with different phenotypes that can contribute to local maladaptation. As in Ronce and Kirkpatrick (2001), immigrants in our system have phenotypes that reflect the populations from which they originate and the rate of successful dispersal depends on the phenotypic distance between the immigrants and the optimum in the recipient pond. Dispersal between each patch is bidirectional, and dispersal rate (*d*) determines the rate at which individuals move passively between ponds and is homogeneous across the metapopulation (symmetric and passive movement).

The change in population size *N* is determined by individual fitness and passive dispersal, and the evolution in the mean phenotype in response to (a)symmetric selection on a single quantitative character is proportional to the product of the additive genetic variance in the character and the intensity of directional selection (Falconer et al., 1996; Lande, 1976), and to the effect of dispersal (Figure S3). At the population level, there is variance around P*_i_* and thus a *demographic load* due to phenotypic variance at the population level. The coupled changes in population size (*N_i_*) (Equation 2a; Figure S2) and phenotype (P*_i_*) (Equation 2b; Figure S3) in each pond *i* are thus expressed as:

(2a) $$\mathrm{Nt}+1\;=\;N_{i}t\;+\;N_{i}t\left[ro\left(1-\frac{N_{i}}{K}\right)-\gamma \left(\frac{\sigma p}{2}\right)-\gamma \left(\frac{{\left({\text{P}}_{i}-\vartheta_{i}\right)}^{2}}{2}\right)+\alpha \gamma \left(\frac{{\left({\text{P}}_{i}-\vartheta_{i}\right)}^{2}}{2}\right)\left({\text{P}}_{i}-\vartheta_{i}+\alpha \right)\left(\vartheta_{i}-{\text{P}}_{i}-\alpha \right)\right]+d\left(\Delta N\right)$$

(2b) $$Pt+1\;={\;\text{P}}_{i}t\;-\;\gamma \left({\text{P}}_{i}-\vartheta_{i}\right)-\alpha \gamma \left({\text{P}}_{i}-\vartheta_{i}\right)\left({\text{P}}_{i}-\vartheta_{i}+\alpha \right)\left(\vartheta_{i}-{\text{P}}_{i}-\alpha \right)+d\left(\frac{N_{i}}{N_{j}}\right)\left(\Delta \text{P}\right)$$

where ∆*N* = (*N_i_* − *N_j_*) and ∆P = (P*_i_* − P*_j_*) represent population size and phenotypic net differences between the two patches respectively (*i* and *j*). As in Ronce and Kirkpatrick (2001), we provide a relative measure of maladaptation *Z*, not limited to integer values, defined as the number of phenotypic standard deviations that separates the phenotype from the environmental optimum in that habitat.

(3) $$Zt=\frac{\left|P_{i}-\vartheta_{t}\right|}{\sigma p}$$

We adopt a definition of maladaptation that is based on the phenotype and that assumes a strict relationship with population size and growth: maladaptation is any phenotypic deviation from the phenotype that maximizes individual fitness (density‐dependent) and equilibrium population size (*N*). By definition, *N* at equilibrium is a decreasing function of *Z* Our simulation results reveal a relationship between local *Z*, regional *Z* (sum over habitats) and ∆*N* (Figure 3). The relationship between trait and fitness is explicit (Equation 1) and because we diagnose phenotype–environment mismatch on a trait space, any deviation from the optimum, even <1*SD* is considered (relative) maladaptation and is quantified on a trait space (acid tolerance) as the trait distance relative to its fitness and abundance maximizing value (Equation 3 and Figure S4). Because the effect of this deviation from the optimum is additive on fitness, it affects the equilibrium (long‐term population size) and will maintain equilibrium population size below the carrying capacity (*K*) as long as there is selection (Ɣ > 0) and as long as (**r > **0). *Z* monotonically increases with the distance (in trait space) of the local population trait from theta (the local environmental optimum) and is summed over local populations to assess regional maladaptation. In our model, per capita (a)symmetric selection strength is density independent and only depends on phenotypic distance from the optimum. However, individual fitness is density‐dependent because it involves intraspecific competition through the carrying capacity (*K*). The net strength of (a)symmetric selection on population growth and mean population phenotype thus depends on density because they are coupled with density‐dependent population growth. All else being equal, fitness, growth, and long‐term (equilibrium) population size are all maximized at the same trait value (the optimum) corresponding to (*Z* = 0). A metapopulation was adapted when maladaptation (*Z* = 0) in both habitats. When *Z* > 0, we considered the (meta)population to be maladapted. We finally implemented stochastic variations in local pH in each pond. At each time step, we added stochastic fluctuations to each local mean pH by drawing random numbers from a normal distribution with zero mean and fixed variance. We implemented spatially independent uncorrelated environmental noise across ponds, as well as positively and negatively correlated pH time series between ponds. All model simulations were run using MATLAB 2016a by MathWorks Inc., Natick, Massachusetts, USA.

## 3 RESULTS

### 3.1 Empirical field experiment

**Figure 2:**
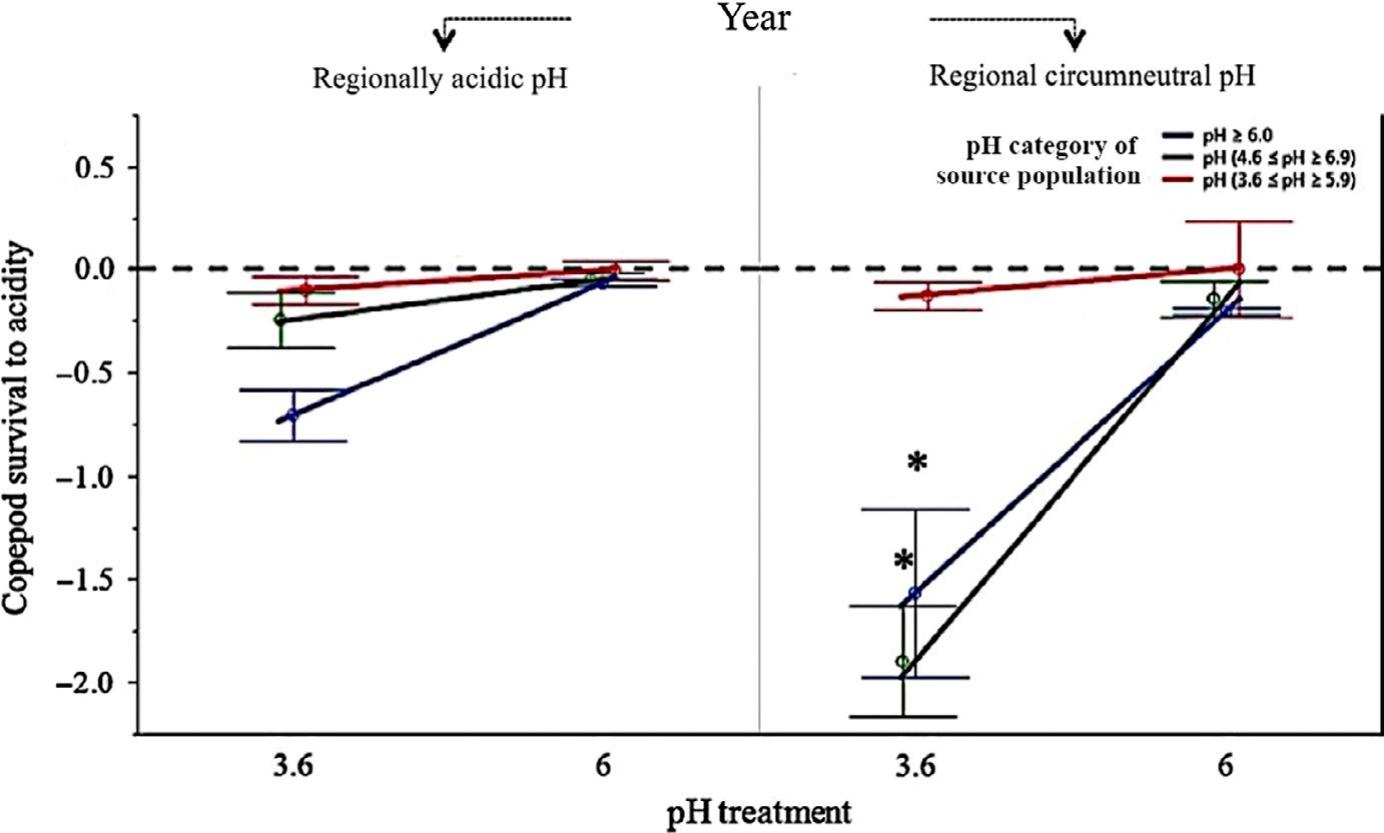
Results of the common garden field experiment conducted during two consecutive years at Cape Race (NL, Canada). The blue line represents the average response of circumneutral pH source copepod populations (*n* = 3 populations), the red line represents the average response of acidic pH source copepod populations (*n* = 3 populations), and the dark green line represents the average response of fluctuating pH source copepods populations (*n* = 3 populations) to two levels of pH (3.6 and 6). Adult *L. minutus* survival to acidity was measured as [Log10 (*N*_final + 1_) ‐ Log10 (*N*_initial + 1_)]. Values above the 0.0 horizontal threshold line indicate increased copepod survival to acidity. Tukey HSD contrasts (*) at alpha = 0.05 represent pairwise differences for the entire model. Error bars represent standard error of the mean (*SEM*)

We did not detect a three‐way interaction between year × copepod population source × pH treatments, but two‐way interactions were detected between most variables (Table 2). Year interacted with copepod population source (*p* = 0.010*, LMM; Table 2) and was indicative of both adaptive and maladaptive phenotype mismatch to environmental pH conditions in the source copepod populations. Indicative of adaptive phenotype–environment mismatch, copepods from local circumneutral ponds (both with stable and fluctuating interannual circumneutral pH) had low acid tolerance and poor adult survival across pH treatments (including exposure to acidic pH 3.6) compared to copepods from acidic ponds (Figure 2). Maladaptive phenotype–environment mismatch was evident in the local circumneutral ponds (both with stable and fluctuating interannual circumneutral pH) in the year when surrounding ponds on the landscape were mostly acidic and the occurrence of other circumneutral ponds was rare (Figure 2). The presence of acid‐tolerant copepod phenotypes from the ponds with stable circumneutral pH in the regionally acidic year was likely indicative of the influence of migration of acid‐adapted copepods from the surrounding landscape. Year strongly interacted with pH treatment exposure to influence copepod adult survival (*p* = 0.0015**, LMM, Table 1). When surrounding ponds on the landscape were more acidic and the frequency of circumneutral habitats was rare, copepods had higher acid tolerance when exposed to acidic pH 3.6, including from ponds that had stable and fluctuating circumneutral pH (Figure 2). There was an interaction between copepod population source and pH (*p* = 0.0026**, LMM; Table 1). Copepods from source ponds with stable acidic pH between years had high acidic tolerance and high adult survival when exposed to both acidic pH 3.6 and circumneutral pH 6.0 (Figure 2). By contrast, copepods from source ponds with stable circumneutral pH between years had low acid tolerance and low adult survival when exposed to acidic pH 3.6 compared to circumneutral pH 6.0 (Figure 2). The asymmetric fitness surface to low pH, between‐pond dispersal of migrant phenotypes, and the outcome on local population trait maladaptation and population size, is explored in our metapopulation model.

**Table 2:** Statistical table including the influence of a covariate and the following main factors

Sources of variation	Nparm	*F* ratio	Prob > F
Initial copepod density [Log10 (*N*_initial + 1_)] (Covariate)	1	38.36	<0.0001***
Year (2013 Acidic vs. 2014 Mildly acidic ‐ circumneutral)	1	12.41	0.0162*
Copepod population source (Acidic vs. Circumneutral vs. Fluctuating)	2	22.12	0.0004***
pH (3.6 vs. 6.0)	1	50.50	<0.0001***
Year × Copepod population source	2	8.53	0.010*
Year × pH	1	19.56	0.0015**
Copepod population source x pH	2	11.68	0.0026**
Year × Copepod population source × pH	2	3.78	0.0612

Note

Factor 1—Year: 2013 (year with acidic regional pond pH); 2014 (year with circumneutral regional pond pH); Factor 2—Category of copepod population source; temporally stable circumneutral pond pH (*n* = 3), temporally stable acidic pond pH (*n* = 3), and fluctuating pond pH (*n* = 3) (see Figure S1); and Factor 3—pH treatment; pH 6.0, pH 3.6 on our response variable, which was final copepod density [Log10 (*N*_final + 1_)] The table includes all two‐way and third‐way interactions from the full factorial model. In the LMM (linear mixed model), we coded individual copepod pond source as a random variable.

By the end of the experiment in both years, final edible chlorophyll *a* algal biomass was abundant across all treatments (Figure S5). In addition, pond temperature was similar between summers based on the temperature data that we collected with (a) point estimates with YSI Pro Plus multi‐parameter water quality meter readings at the onset of the experiment, and (b) continuous temperature readings taken hourly from the start to end of experiment in both years. For the point estimates, at the onset of incubation, mid‐day water surface temperatures were 18.6°C in 2013 and 17.8°C in 2014. For the continuous temperature readings, in 2013 the temperature of incubation was 18.72 ± 0.16 (*N* = 184) and in 2014 the temperature was 18.73 ± 0.17 (*N* = 183).

### 3.2 Two‐patch metapopulation model

#### 3.2.1 No environmental fluctuations

**Figure 3:**
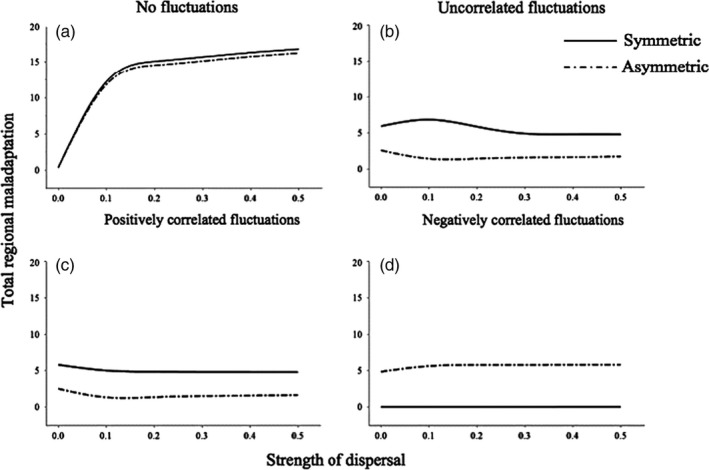
Total maladaptation when the fitness surface for individual phenotypic traits in response to the local environment is symmetric versus asymmetric. (a) Control and stable environmental conditions, (b) uncorrelated environmental fluctuations, (c) positively correlated, and (d) negatively correlated environmental fluctuations. *X*‐axis represents the strength of dispersal and *Y*‐axis the total regional maladaptation (*Z*) to pH in relation to spatial structure and environmental fluctuations, expressed as the sum of local maladaptation values measured in each habitat

**Figure 4:**
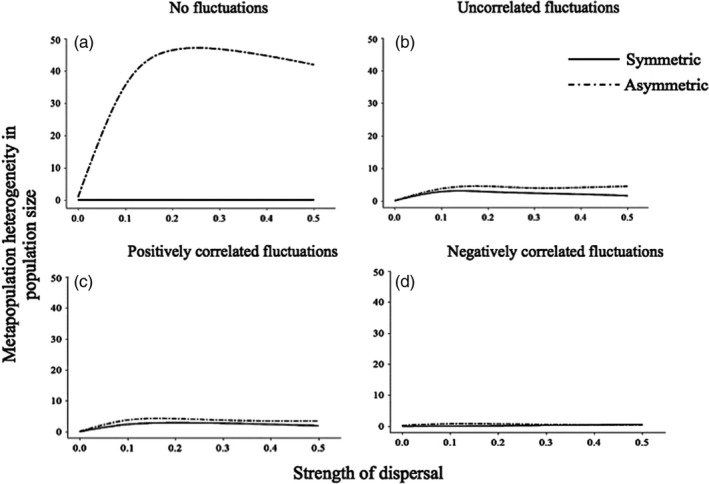
The resulting metapopulation heterogeneity in population size when the fitness surface for individual phenotypic traits in response to the local environment is symmetric versus asymmetric. (a) Control and stable environmental conditions, (b) uncorrelated environmental fluctuations, (c) positively correlated, and (d) negatively environmental correlated fluctuations. *X*‐axis represents the strength of dispersal, and *Y*‐axis represents the metapopulation heterogeneity in population size (*N*)

Under constant pH conditions, regional maladaptation increased with dispersal, but was not affected by selection associated with control parameters (strength and asymmetry) that interact with pH (Figure 3a). The effect of asymmetric selection was instead revealed by the coupled eco‐evolutionary response to dispersal. Increasing dispersal led to strong heterogeneity in equilibrium population sizes between ponds when selection was asymmetric (Figure 4a). Selection strength and asymmetry that interact with pH were required to predict the maintenance of both regional maladaptation and heterogeneous distribution of population abundance between coupled habitats through eco‐evolutionary dynamics.

#### 3.2.2 Uncorrelated, positive, and negative environmental fluctuations

Environmental stochasticity resulted in the maintenance of maladaptation that was robust to dispersal, but also revealed an interaction between the asymmetry imposed directly on the “selection” term of the model, and environmental correlation. Both uncorrelated and correlated stochasticity between ponds resulted in regional maladaptation that was robust to dispersal rate. Asymmetry in the fitness surface to low pH interacted with environmental correlation to determine the level of maladaptation. In the model, asymmetry reduced the level of maladaptation under uncorrelated and positively correlated environment, but was required to produce any maladaptation when pH fluctuations were negatively correlated between ponds. At the metapopulation level and under symmetry and asymmetry acting in the model on fitness, heterogeneity in maladaptation remained weak (Figure 3b). Heterogeneity in population sizes was less important due to the influence of the fluctuations within each patch operating around the deterministic value (Figure 4b). Environmental stochasticity homogenized population sizes across the metapopulation, even in the presence of regional maladaptation, and independently from spatial correlation between environmental fluctuations, and from dispersal rate (Figure 4b–d). Overall, negative spatial correlation in environmental stochasticity between ponds was the only scenario from our simulations leading to a homogeneous distribution of abundance with no maladaptation across all dispersal rate values (Figures 3d and 4d).

## 4 DISCUSSION

We present a first study to explore the interaction between regional selection patterns (spatiotemporal landscape heterogeneity) and limited dispersal on the maintenance of population maladaptation through an asymmetric fitness surface to low pH. While most conservation practices have focused almost exclusively on re‐establishing species and populations that are in decline in terms of abundance and species richness, we highlight the importance of conserving for phenotypic trait variation using trait‐based approaches (Des Roches et al., 2018; Mimura et al., 2017; Salguero‐Gómez, Violle, Gimenez, & Childs, 2018). These approaches can integrate the conservation of ecological health indicators such as population size and species diversity, with the conservation of adaptive potential through phenotypic variation (Hendry et al., 2011; Stockwell, Hendry, & Kinnison, 2003), including local maladaptation (Hendry & Gonzalez, 2008). We defined maladaptation as a phenotype–environment mismatch of acid tolerance in freshwater copepods and provided experimental evidence for the role of interannual differences in regional pH conditions from the surrounding landscape on local maladaptation in copepod populations. Our experimental results further revealed an asymmetric fitness surface to low pH, defined as a dependence of survival on both the magnitude and direction of the population trait deviation from the local pH.

Our model integrated these experimental findings in eco‐evolutionary metapopulation theories by predicting ecological (population size) and evolutionary (maladaptation) responses to spatiotemporal heterogeneity and limited dispersal over regional scales. Under a constant environment, we found that dispersal can interact with asymmetric selection to maintain regional maladaptation and heterogeneous population sizes. We also show the interaction between spatial autocorrelation in environmental fluctuations and asymmetric fitness surfaces on regional maladaptation, despite its decoupling from dispersal and the resulting homogenization of population size. Our findings emphasize the importance of conserving for maladaptation in fragmented populations at the regional scale as adaptive insurance in face of environmental uncertainty over the long term (Derry et al., 2019).

### 4.1 Empirical field experiment

Our field experiment revealed the role of dispersal and regional selection, as well as local (a)symmetric selection, on acid tolerances of copepods in freshwater ponds. We found evidence that dispersal and regional selection determined the pH tolerances of copepods under weak local selection from ponds with temporally stable circumneutral pH, producing phenotype–environment mismatch at the local scale. However, under strong local selection (from ponds with temporally stable acidic pH), a match between acid‐tolerant phenotypes and the acidic environment was maintained despite interannual differences in regional selection. From ponds with temporally fluctuating pH, we found evidence for phenotypic tracking of local pond pH, which was likely reinforced by migration from the surrounding landscape (combined effects of both local and regional selection in the same direction, depending on year). While this finding is congruent with the few other empirical studies that have shown evidence of the combined role of dispersal and landscape context on local adaptive and maladaptive ecological tolerances (Negrín Dastis & Derry, 2016; Tack et al., 2014), our experimental results uniquely demonstrate how the influence of regional selection can depend on the strength of local selection on asymmetric survival responses in local populations.

### 4.2 Two‐patch metapopulation model

We extended existing two‐patch metapopulation models to integrate our experimental findings within a broader eco‐evolutionary theoretical context by predicting the interacting role of limited dispersal and asymmetric survival responses to spatiotemporal variations in (a)symmetric selection strength on ecological (population size) and evolutionary (maladaptation) metrics. Similar to results obtained in another study (Marshall et al., 2010), we also found that maladaptive phenotype–environment mismatch was maximal when selection occurred over spatial scales that are much smaller (within population) than dispersal distance (between population). Our findings are consistent with other theoretical research that has revealed that weak population fitness responses can allow maladaptation to mask phenotypic differences between populations (Cenzer, 2017) or obscure the effects of selection (Bolnick & Nosil, 2007).

Our study shows how maladaptation can be maintained by limited dispersal and suggests that asymmetry in the local fitness surface of the population to phenotypic deviation from the environment optimum is key for explaining the maintenance of heterogeneous population sizes over regional scales when maladaptation is maintained by dispersal. This is because circumneutral‐adapted individuals dispersing into acidic habitats are strongly selected against and do not contribute to changes in population size or to maladaptation. On the contrary, dispersal of acid‐adapted individuals into circumneutral habitats contributes to increases in population size (greater abundance of maladapted phenotypes) due to weak selection for acid tolerance in circumneutral habitat patches. The asymmetric fitness surface to low pH leads to heterogeneous selection strength at the regional scale, thus coupling ecological and evolutionary patterns through the balance between weak selection facilitating local maladaptation, and strong selection promoting optimal growth of local populations.

While spatial heterogeneity in selection strength operates on maladaptation and population abundance, environmental fluctuations can result in the apparent decoupling of evolutionary and ecological responses by homogenizing populations despite the maintenance of maladaptation. Our model results reveal a strong dependence of maladaptation and population size on the interaction between an asymmetric fitness surface and spatiotemporal patterns of selection. Environmental stochasticity generally leads to maladaptation that is robust to the level of dispersal, but that depends instead on the interaction between the fitness surface to low pH (symmetric vs. asymmetric) and spatial correlation in environmental fluctuations (positive vs. negative correlation). Other studies have shown that environmental stochasticity tends to homogenize traits related to spatial dynamics over regional scales (Harrison & Taylor, 1997; Leibold et al., 2004; Mouquet & Loreau, 2003). Our results showed how asymmetry in the individual fitness surface to environmental fluctuations can still drive overall levels of maladaptation despite their robustness to gene flow and despite homogeneous population sizes. These results more generally indicated how the ecological response to environmental variations, and its sensitivity to both asymmetry in the fitness surface to strong selection and dispersal, can lead to an apparent decoupling between ecological and evolutionary metapopulation processes.

The maintenance of heterogeneity in the distribution of species abundance has been extensively studied in ecology (McGill et al., 2007) and can result from differential growth across heterogeneous habitats. However, local adaptation can allow local populations to reach optimal growth, resulting in the homogenization of population abundance through phenotypic tracking of environmental heterogeneity (Edelaar, Jovani, & Gomez‐Mestre, 2017). Our model predicts that an asymmetric fitness surface to local selection can maintain ecological heterogeneity in population abundance over regional scales when maladaptation is maintained by dispersal between environmentally heterogeneous habitats. When applied to the conservation of fragmented landscapes, these results suggest that heterogenous population sizes can be driven by sources of maladapted individuals that inflate both population size and local maladaptation in recipient systems. These emerging source‐recipient systems maintain coupled genetic‐ecological dynamics and heterogeneity. While genetic and population heterogeneity over landscapes are central to regional conservation strategies, including for designing networks of protected areas globally (Rodrigues et al., 2004), they are rarely considered as coupled regional properties, and our study further suggests their sensitivity to environmental fluctuations.

In our field experiment and in our model, the factors affecting the phenotypic distributions and population size dynamics were approximative because we did not account for more specific considerations of the life history of calanoid copepods, such as the existence of a long‐lived resting egg banks of zooplankton (Hairston, 1996). Zooplankton resting egg banks are composed of a mixed variety of phenotypes that depending on local selection at any given point in time, can be a source of maladaptive phenotypes (Rogalski, 2017) or a source of genetic diversity on which selection can act during the process of adaptation (Hairston, 1996).

## 5 CONCLUSION

Our metapopulation model predictions are compatible with the observed maladaptation of local copepod populations to acidic conditions in our field experiment. They further support the role of limited between‐pond dispersal and an asymmetric fitness surface to low pH for the maintenance of maladaptation. Our findings call for a broader eco‐evolutionary theory predicting the role of spatial environmental heterogeneity, dispersal, and (a)symmetric selection in the maintenance of coupled evolutionary (maladaptation) and ecological (abundance distribution) variation at the landscape level. Our study has implications for the conservation of fragmented populations that are challenged by human stressors, as well as for directing attention to the conservation of habitats and populations that could act as sources of adaptive variation across an entire metapopulation within the landscape. Being able to predict expectations for the frequency/persistence of maladaptation in natural systems can inform conservation practitioners of which subpopulations have a large spectrum of adaptive value and or resistance (e.g., tolerance to the stressor) in the face of environmental deterioration (e.g., climate change/environmental fluctuations) (Derry et al., 2019). We emphasize the importance of conserving for maladaptation as the outcome of an ongoing eco‐evolutionary process that has critical implications for the maintenance of biodiversity under changing environmental conditions in the long term.

## CONFLICT OF INTEREST

None declared.

## Data Availability

Data available from the Dryad Digital Repository: https://doi.org/10.5061/dryad.q2j3161 (Negrín Dastis, Milne, Guichard, & Derry, 2019).
