# Supplementary material for: Phenotype–environment mismatch in metapopulations—Implications for the maintenance of maladaptation at the regional scale
Source: Evol Appl. 2019 Jul 25;12(7):1475–86. doi: 10.1111/eva.12833 (PMC6691211; doi:10.1111/eva.12833)
Supplement: Supplementary file 1 [file EVA-12-1475-s001.docx]

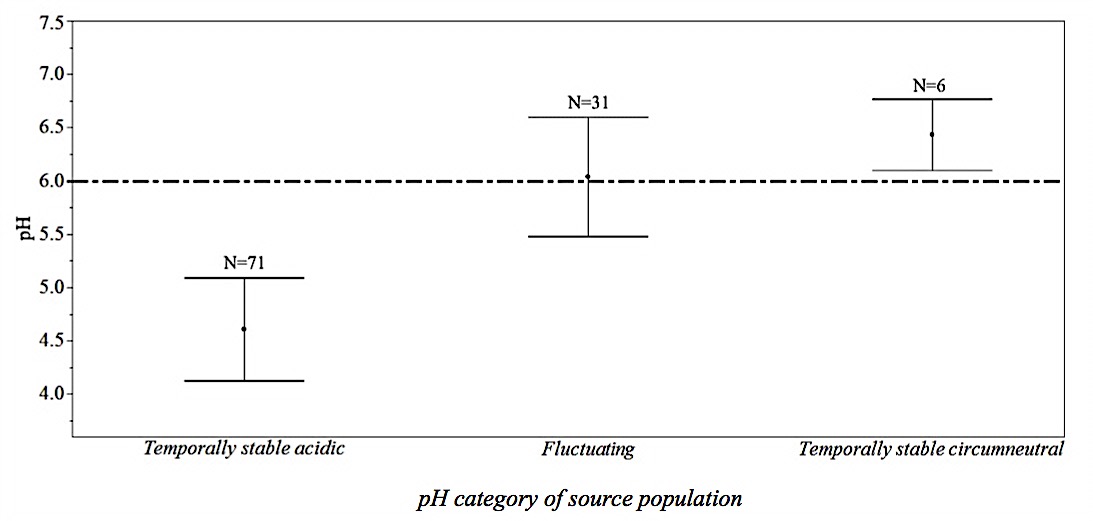


**Figure A1.** pH regime shifts of 108 ponds between three consecutive years (2013, 2014, 2015) at Cape Race (NL, Canada). From left top right, temporally stable acidic environments (N=71), fluctuating pH environments (N=31) and temporally stable circumneutral environments (N=6). This threshold line is crucial to understanding of this graph because it represents the critical biological threshold of acid sensitive crustacean zooplankton species in aquatic systems below which acid sensitive species are lost (Havens et al. 1993, Keller et al. 2002, Holt and Yan 2003). Error bar is constructed using 1 standard deviation from the mean.
