# Supplementary material for: Phenotype–environment mismatch in metapopulations—Implications for the maintenance of maladaptation at the regional scale
Source: Evol Appl. 2019 Jul 25;12(7):1475–86. doi: 10.1111/eva.12833 (PMC6691211; doi:10.1111/eva.12833)
Supplement: Supplementary file 2 [file EVA-12-1475-s002.docx]

**
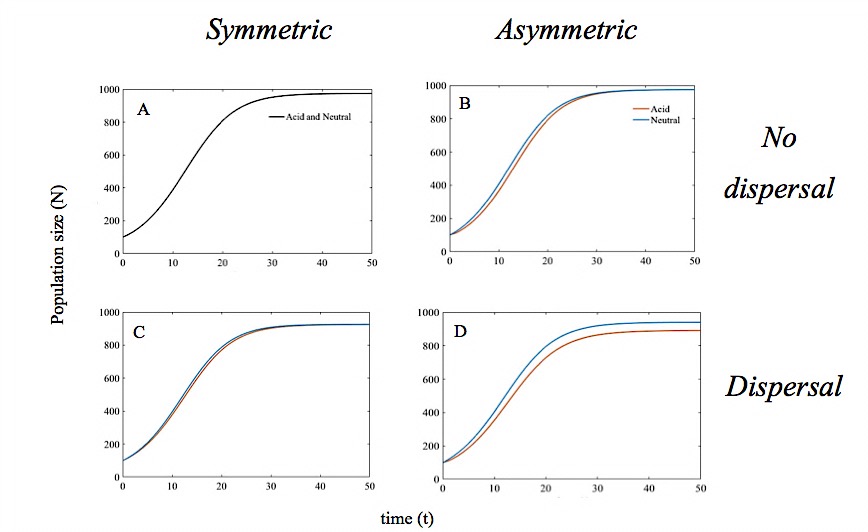
**

**Figure A2.** The influence of weak local selection (Ɣ=0.1) and strong dispersal ($d=0.5)$ on the resulting population size (N) at t=50 for two discrete populations (Acid and Neutral) with symmetric (**A, C**) (alpha=0) and asymmetric (**B, D**) (alpha=0.5) individual phenotypic fitness surfaces. Initial population sizes in both the acid and neutral patch (N=100).
