# Supplementary material for: Phenotype–environment mismatch in metapopulations—Implications for the maintenance of maladaptation at the regional scale
Source: Evol Appl. 2019 Jul 25;12(7):1475–86. doi: 10.1111/eva.12833 (PMC6691211; doi:10.1111/eva.12833)
Supplement: Supplementary file 4 [file EVA-12-1475-s004.docx]

**
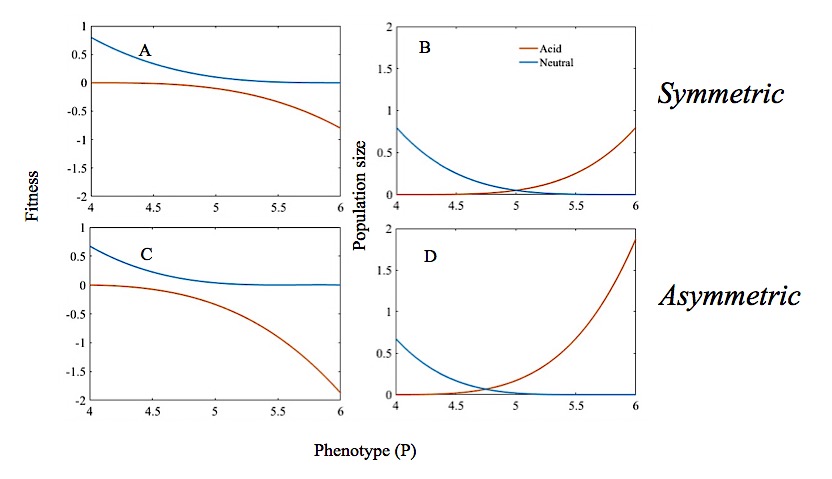
**

**Figure A4.** Model behaviour for values of fitness (**A, C**) and population size in each habitat (**B, D**) under weak selection (Ɣ=0.1) and no dispersal ($d=0)$ for two discrete populations (Acid and Neutral) with symmetric (**A, B**) (alpha=0) and asymmetric (**C, D**) (alpha=0.5) individual phenotypic fitness surfaces to local selection.
