# Supplementary material for: Phenotype–environment mismatch in metapopulations—Implications for the maintenance of maladaptation at the regional scale
Source: Evol Appl. 2019 Jul 25;12(7):1475–86. doi: 10.1111/eva.12833 (PMC6691211; doi:10.1111/eva.12833)
Supplement: Supplementary file 5 [file EVA-12-1475-s005.docx]

**Supplementary methods: common-garden field transplant experiment**

Our field experiment focused on the level of maladaptive acid tolerance in copepod populations from a diversity of pH pond types within the same metapopulation at Cape Race NL, Canada (46°38′33.35′′N, 53°12′02.27′′W). To test for local adaptation and local maladaptation in this fragmented system of isolated ponds, we conducted a common garden experiment in which we transplanted copepod populations (total of n=9) into a common pond environment where they were incubated under common environmental conditions (controlling for temperature, light, food quality and quantity) in closed, translucent 20L bottles that were screened for predators at the onset of the experiment. Pond sources of copepod populations are in close spatial proximity (0.5-1Km^2^), but they are spatially isolated from each other by the landscape matrix composed of a natural carpet of heath moss.

We minimized any possible confounding effects of predation, competition, food and environmental conditions on copepod abundance in the bottles. Copepods were not fed during the experiment, but all bottles contained lake water from another common lake source that included a nutritious phytoplankton community. We measured algal biomass in all experimental bottles, and did not detect any differences in food abundance across pH treatments (Fig. A5). Any predators such as dytiscid beetles, which are the sole invertebrate zooplankton predator in Cape Race ponds (Charette and Derry 2016), were handpicked and removed before placing the copepods in the bottles for the experiment. Bottles were capped to avoid external contamination and included 19L of lake water from a common source and 1L left for water-gas exchange, and there was no water exchange during the experiment. The bottles were incubated at the deepest point (0.5m) of the same pond (Pond Below Q, NL, 46.64897N, -53.21381W).

Copepods were collected from the bottles at the end of the experiment by filtering 19L of pond water through a 54μm mesh and subject to immediate anaesthesia with carbon dioxide exposure, and preservation with buffered 4% sugar-formalin solution. Preservation in 4% sugar-formalin is ideal to preserve changes in biometrical characters and biomass of zooplankton (Haney and Hall 1973). Sexually matured adult copepods were identified with the taxonomic key described by Witty (2004). Following death, copepods decay rapidly in lake or pond water, resulting in visible body fragmentation and deterioration of species morphology. This deterioration is observable under the microscope, and it was used as evidence for low copepod tolerance to acidity because the copepod survival is pH-dependant (see lab experiments done in Derry and Arnott 2007; Negrín Dastis and Derry 2016). Live individuals do not show signs of body decay at the time of sample preservation.

The initial numbers of copepods at the time of inoculation in bottles varied between treatment containers. This initial concentration of copepods in each container was enumerated, [Log_10_ (N_initial + 1_)] transformed, and coded as a covariate in a linear mixed model (LMM) to take these differences in initial starting densities into consideration. An initial minimum copepod abundance sample (N_i_) of 10 adult individuals was also taken and used as a covariate in the model. The pH-dependant response metric was adult copepod survival (N_f_), which was measured as the final numbers of adult copepod individuals present in the sample after 7 days of incubation. N_f_ and N_i_ were Log_10_+1 transformed to improve homogeneity of variance and normality of data. In the mixed model described below, the final number of adult copepod individuals [Log10 (N_ﬁnal + 1_)] was used as the response variable, initial number of individuals [Log10 (N_initial + 1_)] was used as a covariate (Covariate) and pond was used as a random variable in the model. Initial and final numbers of individuals were not pooled together because they were estimated from each container separately.

The experiment had a 2 x 3 x 2 factorial design. The first factor 'Year' occurred on two successive years (two levels: 2013 – summer with acidic regional pH (mean pH 4.76±0.07; n=108 ponds) vs. 2014 – summer with mildly acidic/ close to circumneutral regional pH (mean pH 5.36±0.09; n=108 ponds) (Fig. A1). The second factor 'Copepod population source' had three levels: temporally-stable circumneutral pH ≥ 6.0 ponds (n=3), temporally-stable acidic (3.6 ≤ pH ≥ 5.9) ponds (n=3), and fluctuating pH (4.6 ≤ pH ≥ 6.9) ponds (n=3). The third factor 'pH treatment' had two levels: pH 6.0 vs. pH 3.6. The level of replication in the experiment had a balanced n=3 for each treatment combination, resulting in a total of 36 experimental units. All tests were run at a significant value of α=0.05. Based on the response variable residuals and a goodness of fit test, the data is from the Normal distribution (AICc 45.92; Shapiro–Wilk W test; 0.95 Prob < W 0.17). The full factorial model was analysed with a linear mixed factorial model (LMM) with the lme4 package in R (R Core Team, 2016), in which the three fixed factors are described above, and pond identity was included as a random variable.

Additional details for implementing the field experiment are described for a different experiment with similar methods that was conducted by Negrín Dastis and Derry (2016).

**References**

Charette, C. and Derry, A.M., 2016. Climate alters intraspecific variation in copepod effect traits through pond food webs. *Ecology*, *97*, 1239–1250.

Derry, A.M., Arnott, S.E. (2007). Adaptive reversals in acid tolerance in copepods from lakes recovering from historical stress. *Ecological Applications*, *17*, 1116–1126.

Haney, J.F., Hall, D.J. (1973). Sugar‐coated *Daphnia*: A preservation technique for Cladocera. *Limnology and Oceanography*, *18*, 331–333.

Negrín Dastis, J.O., Derry, A.M. (2016). The impact of regional landscape context on local maladaptive trait divergence: a field test using freshwater copepod acid tolerance. *Evolutionary Ecology*, *30*, 841–859.

R Core Team. (2016). R: A Language and Environment for Statistical Computing. R Foundation for Statistical Computing, Vienna, Austria.

Witty, L.M. (2004) Practical Guide to Identifying Freshwater Crustacean Zooplankton. 2nd Edition, Cooperative Freshwater Ecology Unit, Sudbury, 50 p.
